# Partial Replacement of Diet with Dehulled Adlay Ameliorates Hepatic Steatosis, Inflammation, Oxidative Stress, and Gut Dysbiosis in Rats with Nonalcoholic Fatty Liver Disease

**DOI:** 10.3390/nu15204375

**Published:** 2023-10-16

**Authors:** Hsuan-Chih Huang, Pei-Ni Lee, Wen-Chih Huang, Hsin-Yi Yang

**Affiliations:** 1 Department of Nutritional Science, Fu Jen Catholic University, No. 510, Zhongzheng Rd., Xinzhuang District, New Taipei City 24205, Taiwan; 2 Department of Nutrition, Taipei Hospital, Ministry of Health and Welfare, No. 127, Siyuan Rd., Xinzhuang District, New Taipei City 24250, Taiwan; diet10096@tph.mohw.gov.tw; 3 Department of Anatomical Pathology, Taipei Institute of Pathology, No. 146, Sec. 3, Chongqing N. Rd., Datong District, Taipei City 10374, Taiwan

**Keywords:** nonalcoholic fatty liver disease, dehulled adlay, steatosis, inflammation, oxidative stress, gut microbiota

## Abstract

The prevalence of nonalcoholic fatty liver disease (NAFLD) has been increasing worldwide, and the average age at NAFLD diagnosis has been decreasing. Although some components of adlay can ameliorate lipid metabolism, oxidative stress, inflammatory response, and gut microbiota, few studies have explored the effects of the dietary intake of intact dehulled adlay on liver diseases. Therefore, in this study, we investigated the effects of the dietary intake of dehulled adlay on NAFLD progression and explored the potential underlying mechanisms. Rats were randomized into a control group; a high-fat, high-sucrose diet (60% total energy derived from fat and 9.4% from sucrose)-induced NAFLD group (N); or a high-fat, high-sucrose diet with dehulled adlay group (received the same amounts of dietary fiber and total energy as did the N group). The experimental duration was 16 weeks. The diet containing dehulled adlay mitigated hepatic fat accumulation, proinflammatory cytokine levels, and oxidative stress by regulating the AMPK-Nrf2-NLRP3 inflammasome pathway and ferroptosis. Additionally, the dietary intake of dehulled adlay modulated the composition of the gut microbiota. In conclusion, a diet containing dehulled adlay may decelerate the progression of NAFLD by ameliorating hepatic steatosis, inflammation, oxidative stress, and gut dysbiosis.

## 1. Introduction

Nonalcoholic fatty liver disease (NAFLD) is the abnormal accumulation of fat in the liver mainly because of unhealthy dietary habits, not including excessive alcohol intake. The global prevalence of NAFLD is approximately 25% and has been increasing rapidly, and the average age at NAFLD onset has been declining [1]. However, the pathogenesis of NAFLD remains unclear. The multiple-hit hypothesis, which is the most widely accepted hypothesis in this context, suggests that NAFLD results from the interaction of many risk factors, such as oxidative stress, inflammation, and gut dysbiosis [1]. Ferroptosis also drives the progression of NAFLD by affecting oxidative stress and the inflammatory response [2]. Dietary phytochemicals and polysaccharides may play regulatory roles in hepatic pathologies, but additional in vivo evidence is required to clarify their roles [3]. If NAFLD is not treated properly in its early stages, it may progress from fatty liver to steatohepatitis or even irreversible fibrosis and cirrhosis [1,3]. According to a report by the American Association for the Study of Liver Diseases, no specific medication is available for treating NAFLD, and the most useful treatment strategies focus primarily on lifestyle-based interventions, including exercise and dietary modifications [4,5]. Therefore, identifying appropriate dietary practices for managing this disease is of utmost importance.

Whole grains contain more nutrients and bioactive compounds with antioxidative and anti-inflammatory properties than do refined grains, thus offering additional benefits for human health [6]. Dietary fibers derived from cereals have been reported to exhibit stronger anti-inflammatory properties than those derived from fruits and vegetables [7]. The Dietary Guidelines of Taiwan recommend that one-third of the daily intake of food should comprise unrefined whole grain cereals as a staple food to reduce the prevalence of chronic diseases. Adlay is a common dietary grain in Asia and is used for therapeutic purposes in traditional medicine. It exhibits considerable potential for regulating oxidative stress, inflammation, and lipid metabolism [8]. An in vivo study revealed that adlay bran extract can serve as a botanical supplement to improve hyperlipidemia and induce the expression of AMP-activated protein kinase (AMPK) in hamsters consuming a high-fat diet [9]. An in vitro study demonstrated that the dietary intake of adlay seed extract downregulated the expression of tumor necrosis factor α (TNF-α) and interleukin (IL)-6 by inhibiting the nuclear factor-κB (NF-κB) and NLR family pyrin domain containing 3 (NLRP3) inflammasome pathway in lipopolysaccharide (LPS)-induced RAW 264.7 macrophages [10]. In HepG2 cells, adlay seed polyphenol extract increased antioxidative enzyme expression and reduced oxidative stress by modulating the nuclear factor erythroid 2-related factor 2 (Nrf2) pathway under H_2_O_2_-induced oxidative stress [11]. Li et al. indicated that adlay bran polysaccharide extract improved gut barrier function by inhibiting NF-κB protein expression in Caco-2 cells with TNF-α-induced epithelial barrier dysfunction [12].

Unlike refined adlay, native adlay in Taiwan is often sold as dehulled adlay. It contains not only the adlay seed but also its bran, which may be the most functionally bioactive part of the grain [13]. Adlay may lose some nutrients and bioactive substances during its processing for the synthesis of other products [14]. Thus, the inclusion of unprocessed dehulled adlay in daily meals may be the most healthy and practical approach for incorporating this grain into dietary habits and lifestyles. Although many studies have reported the potential benefits of adlay in various metabolic disorders, most have used adlay extract as a complementary therapy in in vitro models to elucidate the underlying mechanisms. Further research is necessary to comprehensively analyze the effects of consuming the intact form of dehulled adlay on the progression of NAFLD. Therefore, in this study, we investigated the effects of the dietary intake of dehulled adlay on NAFLD progression and explored the underlying mechanisms. The amounts of dietary fibers and macronutrients in the diets were adjusted to exclude the possible effects of these factors and mimic the substitution of dietary components with dehulled adlay.

## 2. Materials and Methods

### 2.1. Sample Preparation

Dehulled adlay (Taichung No. 5) was kindly provided by the Taichung District Agricultural Research and Extension Station (Taichung, Taiwan). Dehulled adlay (100 g) consists primarily of carbohydrates (61.7 g), protein (17.6 g), lipids (7.6 g), and dietary fiber (1.7 g). This grain was steamed for 30 min, dried, and ground to powder as previously described [14]. The levels of total polyphenols and total polysaccharides in the prepared sample were determined using the Folin–Ciocalteu method [15] and the phenol-sulfuric acid method [16]. The levels of total polyphenols and total water-soluble polysaccharides in the sample powder were 9.5 mg/gallic acid equivalents/100 g and 21.5%, respectively.

### 2.2. Animals and Experimental Design

Seven-week-old Sprague–Dawley rats were purchased from BioLASCO, Taiwan. The rats were housed in the Experimental Animal Center per the guidelines of the Institutional Animal Care and Use Committee of Fu Jen Catholic University (approval ID: 109035). They were maintained in an environment with a constant temperature (23 °C ± 2 °C) and humidity (55% ± 10%) and were subjected to a 12-hour light/dark cycle in accordance with the Animal Protection Act and the regulations of the Ministry of Agriculture, Executive Yuan. They were fed with a standard rat chow diet (Rodent Autoclavable Laboratory Chow 5010, Purina Mills, St. Louis, MO, USA) for 1 week for adaptation. Furthermore, the rats were randomized into three groups (*n* = 8): a control group (C), a diet-induced NAFLD group (N), and an NAFLD plus dehulled adlay group (NA). Group C was fed a normal chow diet (13.5% total energy from fat, 58.2% from carbohydrate, and 1% from sucrose). Groups N and NA were fed with a high-fat, high-sucrose (HFHS) diet (60% total energy derived from fat, 20% from carbohydrate, and 9.4% from sucrose). However, the diet of Group NA contained dehulled adlay powder, which was substituted for components of the HFHS diet to maintain equal intake amounts of macronutrients and fibers between the groups. The diet compositions are listed in Supplementary Table S1. During the 16-week experimental period, food and water were provided ad libitum. The rats’ energy intake was calculated daily, and their body weight was recorded weekly. At the end of the experiment, fresh rat feces were collected for the analysis of the gut microbiota. Subsequently, the rats were euthanized to obtain blood and liver samples for further analysis.

### 2.3. Blood and Tissue Sampling and Analysis

#### 2.3.1. Blood Sample Analysis

Blood samples were collected and centrifuged at 1500× *g* for 15 min at 4 °C to obtain serum samples, which were used to measure the levels of aspartate aminotransferase (AST), alanine aminotransferase (ALT), triglyceride (TG), total cholesterol (TC), high-density lipoprotein cholesterol (HDL-C), and low-density lipoprotein cholesterol (LDL-C) levels by using a chemical analyzer (Modular P800 Autoanalyzer, Roche Diagnostics, Basel, Switzerland). Commercial kits were used to analyze the levels of endotoxin (A39553, Thermo, Rockford, IL, USA) and FFA (FA115, Randox, Crumlin, UK) per the manufacturers’ instructions.

#### 2.3.2. Liver Sample Analysis

After the livers were perfused with 0.9% saline, samples were collected and stored at −80 °C. Liver lipids were extracted using chloroform and methanol, as previously described [17]. The hepatic levels of TG (TR210, Randox, Crumlin, UK), TC (BXC0271, Fortress, Antrim, UK), and FFA (FA115, Randox, Crumlin, UK) were measured using diagnostic kits. To determine the hepatic levels of proinflammatory cytokines, glutathione (GSH)/glutathione reductase (GSSG), and malondialdehyde (MDA), the samples were homogenized in buffer containing 130 mM NaCl, 2.6 mM KCl, 8 mM Na_2_HPO_4_·2H_2_O, 1.5 mM KH_2_PO_4_, 2 mM ethylenediaminetetraacetic acid, and protease inhibitors. After centrifugation, the supernatants were stored at −80 °C. The hepatic levels of TNF-α and IL-1β were measured using enzyme-linked immunosorbent assay kits (DY-410-05 and DY510-05, R&D Systems, Minneapolis, MN, USA). The levels of hepatic GSH and GSH/GSSG were measured using a commercial GSH assay kit (703002, Cayman Chemical, Ann Arbor, MI, USA). The level of hepatic MDA was measured using the thiobarbituric acid reactive substances test, as described by Gonzalez Flecha et al. [18].

For Western blotting, liver samples were homogenized in a buffer containing 50 mM Tris-HCl, 150 mM NaCl, 0.1% sodium dodecyl sulfate, 1% NP-40 (pH 7.5), and protease inhibitors. The homogenate was centrifuged at 10,000× *g* for 15 min at 4 °C. After centrifugation, the supernatant was stored at −80 °C. For the analyses of Nrf2 and NF-κB, liver samples were homogenized in a buffer containing 50 mM Tris–HCl, 150 mM NaCl, 0.1% sodium dodecyl sulfate, 1% NP-40 (pH 7.5), and phosphatase inhibitors; the nuclei were extracted using a commercial nuclear extraction kit (ab289882, Abcam, Cambridge, UK). Western blotting was performed to measure the levels of phosphorylated AMPK (pAMPK), AMPK, peroxisome proliferator-activated receptor α, very low-density lipoprotein (VLDL) receptor (VLDLr), sterol regulatory element-binding transcription factor 1 (SREBP1), Nrf2, NF-κB, NLRP3, cysteine-dependent aspartate-specific protease 1 (caspase 1), caspase 1–20 kDa (p20), toll-like receptor 4 (TLR4), TIR-domain-containing adaptor-inducing interferon-β (TRIF), myeloid differentiation primary response 88 (MyD88), ferroportin (FPN), heme oxygenase 1 (HO-1), and nuclear receptor coactivator 4 (NCOA4) using appropriate antibodies followed by corresponding horseradish peroxidase–conjugated secondary antibodies. Samples containing 30 μg of protein were separated on a 10% sodium dodecyl sulfate-polyacrylamide gel and transferred onto a nitrocellulose membrane (IPVH00010, MilliporeSigma, Burlington, MA, USA). Nonspecific binding sites were blocked through the overnight incubation of the membranes at 4 °C in 5% nonfat milk or a commercial blocking buffer. After washing with phosphate-buffered saline/Tween-20, the membranes were incubated with primary antibodies at room temperature for 1 or 2 h. After being washed with PBS/Tween-20, membranes were incubated with a rabbit rabbit pAMPK (2532, Cell Signaling, MA, USA), AMPK (2325S, Cell Signaling, MA, USA), rabbit SREBP1 (GTX79299, Gentex, MI, USA), rabbit PPARα (ab8934, Abcam, Cambridge, UK), rabbit VLDLr (19493-1-AP, Proteintech, IL, USA), rabbit NF-κB (3033, Cell Signaling, MA, USA), rabbit Nrf2 (16396-1-AP, Proteintech, IL, USA), rabbit NLRP3 (ab263899, Abcam, MA, USA), rabbit caspase 1 (AF5418, Affinity Bioscience, OH, USA), rabbit caspase1 (p20) (AF4005, Affinity Bioscience, OH, USA), TLR4 (19811-1-AP, Proteintech, IL, USA), rabbit TRIF (NB120-13810, Nouvous, CO, USA), rabbit MyD88 (4283S, Cell signaling, MA, USA), NCOA4 (DF4225, Affinity Bioscience, OH, USA), or rabbit FPN (AF4727, Affinity Bioscience, OH, USA) at room temperature for 2 or 1 h followed by incubation with an HRP-conjugated anti-mouse antibody (405306, Biolegend, CA, USA) and anti-rabbit antibody (440640, Biolegend, CA, USA) for second antibody. Membranes were then washed, and the immune complex was developed using a chemiluminescence detection system (Western Lighting Plus-ECL, PerkinElmer, MA, USA). Equal total protein loading was verified using a commercially available mAb against GAPDH or PCNA.

Rat liver tissues were fixed in formaldehyde for histological analysis. The samples were stained with hematoxylin–eosin. A blinded pathologist examined the biopsy samples. The liver was observed at 200× magnification for the evaluation of fatty change, and the degree of hepatic fatty change was assessed and scored by a pathologist [19].

#### 2.3.3. Gut Microbiota Analysis

Fresh fecal samples were collected before euthanizing the rats. Fecal microbial DNA was extracted using a method described by Godon et al. [20], and 16S rRNA gene sequencing was performed following a method described by Zhou et al. [21]. The sequence with the highest frequency in an operational taxonomic unit (OTU), according to the algorithm used, was considered to be the representative sequence of that out. The α-diversity index can be used to evaluate the richness and diversity of microbial communities in a sample group and the evenness of their distribution. For differential analysis and comparison of microbial community composition between different samples, β-diversity is used [22]. The RDP Classifier (v2.11) was used to perform taxonomic classification of the representative sequences of the OTUs (confidence threshold value: 0.8) and divide them by taxonomic level: kingdom, phylum, class, family, and genus [23].

### 2.4. Statistical Analysis

Data are presented in terms of the mean ± standard error of the mean values. Statistical analyses were performed using SAS (version 9.3; SAS Institute, Cary, NC, USA). Data recorded at the end of the experiment were compared among the groups through one-way analysis of variance, followed by Tukey’s multiple comparison test (post hoc validity test). Spearman’s correlation analysis was performed to analyze the correlations between different variables and the gut microbiota. A *p* value of <0.05 indicated statistical significance.

## 3. Results

### 3.1. Effects of Dehulled Adlay on Modulating HFHS Diet-Induced NAFLD

**Figure 1:**
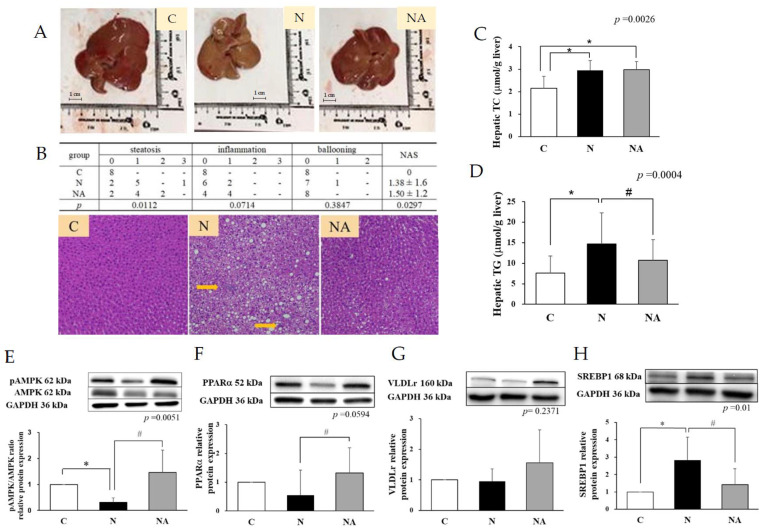
Hepatic fat accumulation and lipid metabolism-related protein expression of rats at the end of the experiment. (**A**) Liver tissue appearance. (**B**) Hematoxylin–eosin staining results of liver biopsy and pathological scores. Levels of (**C**) hepatic TC, (**D**) hepatic TG, and relative protein expression of (**E**) phosphorylated AMPK/AMPK ratio, (**F**) PPARα, (**G**) VLDLr, and (**H**) SREBP1. Data are presented in terms of the mean ± standard error of mean values; *n* = 6. One-way analysis of variance was followed by Tukey’s multiple comparison test. C, control diet; N, nonalcoholic fatty liver disease diet; NA, nonalcoholic fatty liver disease diet with dehulled adlay. The nonalcoholic fatty liver disease activity score was calculated in terms of steatosis (0–3), lobular inflammation (0–3), and hepatocellular ballooning (0–2). TC, total cholesterol; TG, total glyceride; AMPK, AMP-activated protein kinase; PPARα, peroxisome proliferator-activated receptor alpha; SREBP1, sterol regulatory element-binding transcription factor 1; VLDLr, very low-density lipoprotein receptor; yellow arrow, ballooning. * *p* < 0.05, compared with Group C; ^#^ *p* < 0.05, compared with Group N.

After the 16-week experiment, the mean daily intake of dehulled adlay in Group NA was 5.5 ± 1.0 g/kg body weight. The energy intake did not vary significantly among the groups. Thus, differences in caloric intake may be excluded as a cause of the observed effects. Furthermore, no significant intergroup differences were noted in the changes in body weight or the liver weight/body weight ratio (Table 1). Although the serum levels of AST, ALT, and TG in Group N and Group NA were significantly lower than those in Group C, the levels of FFA, TC, HDL-C, LDL-C, or HDL-C did not vary significantly among the groups. Group N exhibited a higher level of fat accumulation in the liver than did the other groups, as demonstrated by hematoxylin-eosin staining. Furthermore, Group N had an NAFLD activity score of 1.38 (Figure 1B), suggesting mild steatosis; this finding indicates the successful establishment of the NAFLD model. Regarding hepatic proinflammatory cytokines, Group N exhibited a significantly higher level of IL-1β and an insignificantly higher level of TNF-α than did Group C and Group NA (Table 1).

### 3.2. Dehulled Adlay Ameliorated HFHS Diet-Induced Liver Steatosis by Modulating β-Oxidation and De Novo Lipogenesis

At the end of the experiment, the hepatic levels of TC and TG were higher in Group N than in Group C. Group NA had a significantly lower level of TG than did Group N (Figure 1C,D). The hepatic pAMPK/AMPK ratio and PPARα expression level were lower, but the hepatic SREBP1c expression level was higher in Group N than in Group C and Group NA. Group NA exhibited a higher expression level of VLDLr than did Group N; however, this trend was nonsignificant (Figure 1E–H).

### 3.3. Dehulled Adlay Ameliorated HFHS Diet-Induced Oxidative Stress by Upregulating Nrf2, Antioxidant Systems, and Ferroptosis

**Figure 2:**
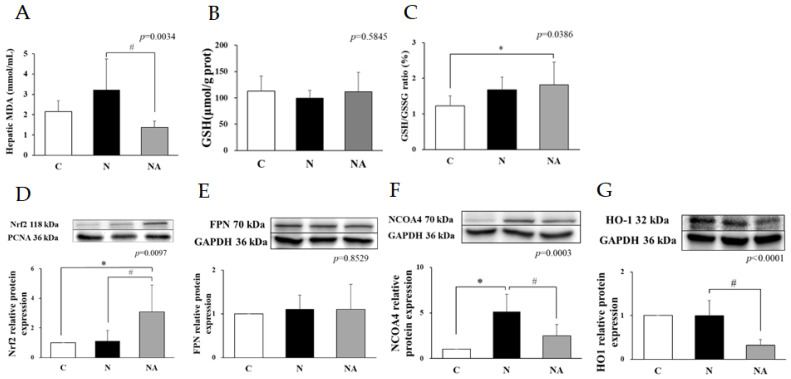
Hepatic MDA, glutathione, and oxidative stress-related protein expression of rats at the end of the experiment. (**A**) Hepatic MDA levels; (**B**) total GSH levels; (**C**) GSH/GSSG ratio; and the relative protein expression levels of (**D**) Nrf2, (**E**) FPN, (**F**) NCOA4, and (**G**) HO-1. Data are presented in terms of the mean ± standard error of mean values; *n* = 6. One-way analysis of variance was followed by Tukey’s multiple comparison test. Lower-case letters denote significantly different data groups in each panel. C, control diet; N, nonalcoholic fatty liver disease diet; NA, nonalcoholic fatty liver disease diet with dehulled adlay; MDA, malondialdehyde; GSH, glutathione; GSSG, glutathione; Nrf2, nuclear factor erythroid 2-related factor 2; NCOA4, nuclear receptor coactivator 4; FPN, ferroportin; HO-1, heme oxygenase 1. * *p* < 0.05, compared with Group C; ^#^ *p* < 0.05, compared with Group N.

Although the level of total GSH did not vary significantly among the groups, Group N had a nonsignificantly higher GSH level than did the other groups. Group NA had a significantly lower hepatic MDA level than did Group N (Figure 2A,B). Furthermore, Group NA had a higher GSH/GSSG ratio than did Group C (Figure 2C). Western blotting revealed that the expression level of Nrf2 was higher in Group NA than in the other groups (Figure 2D). Regarding ferroptosis-related proteins, the expression level of NCOA4 was higher in Group N than in Group C, and the expression levels of both HO-1 and NCOA4 were lower in Group NA than in Group N. FPN levels did not vary significantly among the groups (Figure 2E–G).

### 3.4. Dehulled Adlay Ameliorated HFHS Diet-Induced Inflammation by Downregulating the NLRP3 Inflammasome Pathway

**Figure 3:**
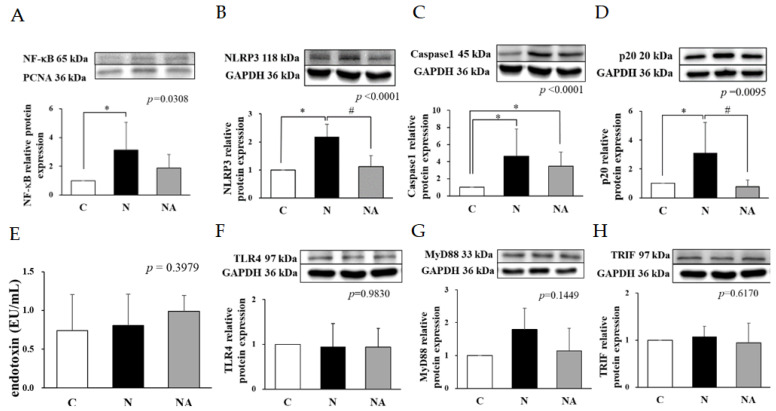
Hepatic inflammation-related protein expression and endotoxin levels of rats at the end of the experiment. (**A**) NF-κB, (**B**) NLRP3, (**C**) caspase1, (**D**) p20, (**E**) endotoxin, (**F**) TLR4, (**G**) MyD88, and (**H**) TRIF. Data are presented in terms of the mean ± standard error of mean values; *n* = 6. One-way analysis of variance was followed by Tukey’s multiple comparison test. C, control diet; N, induced NAFLD diet; NA, induced NAFLD diet substitute adlay. NF-κB, nuclear factor-κB; NLRP3, NOD-, LRR-, and pyrin domain-containing protein 3; Casepase1, cysteine-containing asparte-specific protease; TLR4, toll-like receptor 4; MyD88, myeloid differentiation primary response protein 88; TRIF, TIR-domain-containing adapter-inducing interferon-β * *p* < 0.05 compared with group C; # *p* < 0.05 compared with group N.

The hepatic levels of cytokines were higher in Group N than in the other groups (Table 1). Therefore, we analyzed the expression levels of related proteins. The hepatic expression levels of NF-κB, NLRP3, caspase 1, and p20 were higher in Group N than in Group C but lower in Group NA than in Group N (Figure 3A–D). The levels of endotoxin and the relative expression levels of TLR4, MyD88, and TRIF did not vary significantly among the groups (Figure 3E–G).

### 3.5. Dehulled Adlay Ameliorated HFHS Diet-Induced Gut Dysbiosis by Modulating Gut Microbiota Abundance

**Figure 4:**
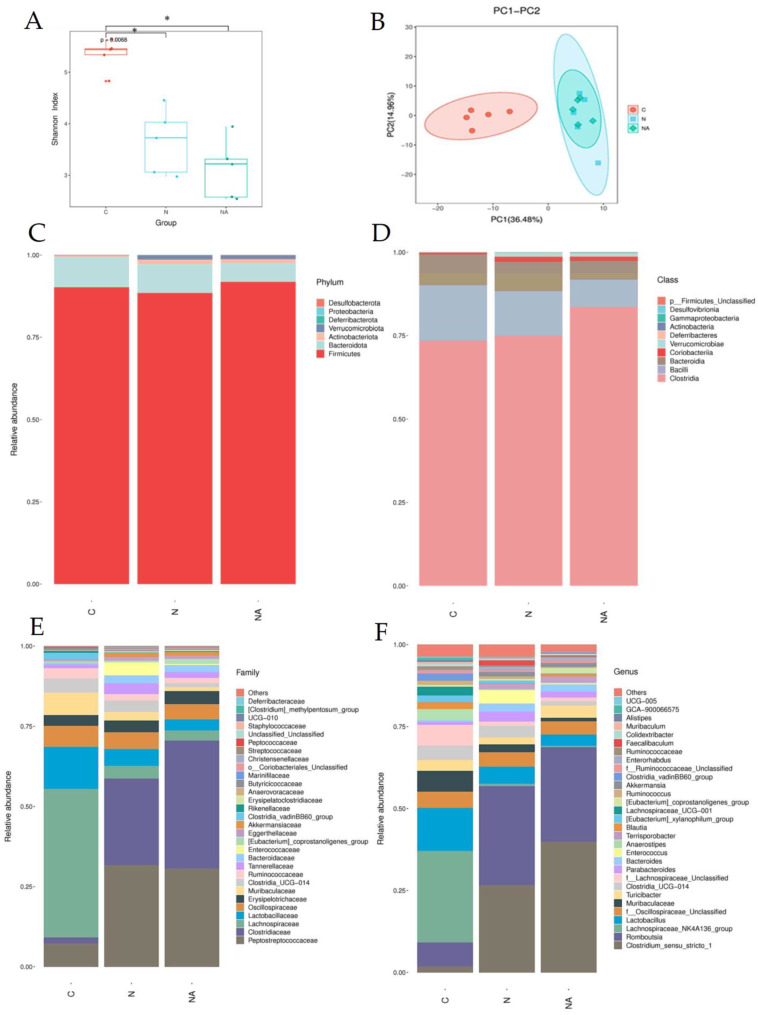
Fecal microbiota analysis of rats at the end of the experiment. (**A**) Shannon index values, (**B**) PCA analysis results, and the relative abundances of organisms belonging to different (**C**) phyla, (**D**) classes, (**E**) families, and (**F**) genera. Data are presented in terms of the mean ± standard error of mean values; *n* = 5. One-way analysis of variance was followed by Tukey’s multiple comparison test. C, control diet; N, nonalcoholic fatty liver disease diet; NA, nonalcoholic fatty liver disease diet with dehulled adlay; PCA, principal component analysis. * *p* < 0.05 compared with Group C.

**Figure 5:**
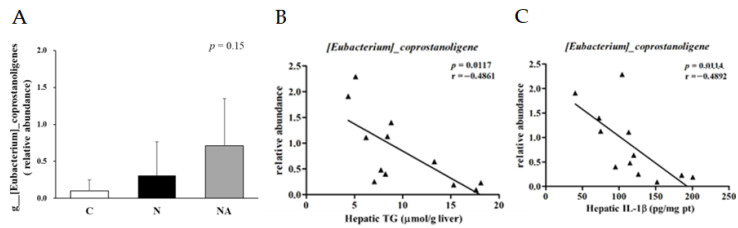
(**A**) Relative abundance of *Eubacterium coprostanoligenes* in each group and the correlation between the abundance of *E. coprostanoligenes* and the levels of (**B**) hepatic total glycerides and (**C**) interleukin-1β. Data are presented in terms of the mean ± standard error of mean values; *n* = 4. One-way analysis of variance was followed by Tukey’s multiple comparison test. C, control diet; N, nonalcoholic fatty liver disease diet; NA, nonalcoholic fatty liver disease diet with dehulled adlay.

We analyzed the composition of gut microbiota in fresh rat fecal samples to understand the effects of dehulled adlay consumption on gut health. In terms of the Shannon index, both Group N and Group NA exhibited lower α-diversity than did Group C (Figure 4A). Principal component analysis indicated that Group N and Group NA were similar in terms of the distribution of gut microbiota; however, both groups differed from Group C in this context (Figure 4B). Nevertheless, further classification of the gut microbiota revealed some intergroup differences (Figure 4C–F). At the family level (Figure 4E), the relative abundances of *Peptostreptococcaceae* and *Clostridiaceae* were higher, and those of *Lachnospiraceae* and *Lactobacillaceae* were lower in Group N and Group NA than in Group C. To compare Group N and Group NA, we conducted genus-level analyses (Figure 4F). The relative abundances of *Clostridium_sensu_stricto_1* and *Erysipelatoclostridium* sp. were higher than those of *Peptococcaceae* and lower in Group NA than in Group N. The relative abundance of *Eubacterium coprostanoligenes* was the highest in Group NA, where it was the predominant bacterial species (Figure 5A). The abundance of this bacterium correlated negatively with the hepatic levels of TG and IL-1β (Figure 5B,C).

## 4. Discussion

In the present study, we fed rats an HFHS diet for 16 weeks. Subsequent histological and quantitative analyses revealed significant hepatic accumulation of TG, indicating the successful establishment of NAFLD. The changes in body weight did not vary between the control rats and experimental rats; this might have been because they had similar caloric intakes. When studying patients with NAFLD, Xu et al. suggested that patients without obesity have lower levels of AST and ALT than do those with obesity [24]; this observation may explain our finding pertaining to the lower levels of AST and ALT in Group N than in Group C. We observed a greater amelioration of hepatic steatosis and inflammation in Group NA than in Group N. These findings suggested that dehulled adlay decelerates the progression of NAFLD.

The liver is the main organ that modulates lipid metabolism in vivo, and the imbalance of lipid synthesis and catabolism leads to the abnormal accumulation of fat in the liver [25]. In the present study, Group N exhibited a lower serum level of TG but a higher level of hepatic fat accumulation than did the other groups. Group N also exhibited a lower level of AMPK activation, a higher level of SREBP1c expression, and a lower level of PPARα expression than did the other groups. An imbalance in de novo lipogenesis and lipid oxidation may cause hepatic steatosis. VLDL removes fatty acids from the hepatic tissue, and the rate of VLDL secretion linearly correlates with the hepatic level of TG under physiological conditions [25]. By contrast, in patients with NAFLD, the rate of VLDL secretion plateaus, potentially leading to the hepatic accumulation of VLDL–TG and the reduction of serum TG levels [26]. VLDL is metabolized to LDL more easily if it contains more cholesterol during lipoprotein metabolism [27]. Group N exhibited insignificantly lower expression levels of VLDLr and higher serum levels of TC and LDL-C than did Group NA. This may be explained by lipoprotein metabolism; extending the experimental period may help elucidate the underlying mechanisms. We further noted that a diet containing dehulled adlay inhibits hepatic steatosis by modulating β-oxidation and de novo lipogenesis without directly causing weight loss.

Oxidative stress accelerates the progression of NAFLD [1]. The activation of Nrf2 upregulates the antioxidative system to suppress oxidative stress [28]. Group NA exhibited a higher expression level of Nrf2 and a lower hepatic level of MDA than did Group N and a higher GSH/GSSG ratio than did Group C. GSH can reduce oxidative stress through reduction–oxidation reactions with enzymes [28]. We found that the dietary intake of dehulled adlay modulated the expression of ferroptosis-related proteins. Ferroptosis, the accumulation of iron, potentially increases the level of MDA and leads to an imbalance in GSH metabolism [29]. It may also accelerate the progression of NAFLD by increasing oxidative stress and proinflammatory responses [2]. We noted no significant intergroup difference in the expression level of FPN, which facilitates the release of Fe^2+^ from the liver; however, Group N had higher levels of HO-1 and NCOA4, which potentially increase Fe^2+^ release and lead to iron accumulation in the liver, than did the other groups. By contrast, Group NA exhibited lower expression levels of these proteins than did Group N, indicating the efficacy of dehulled adlay in preventing oxidative damage.

The progression of NAFLD may be accelerated by gut dysbiosis [26], which likely facilitates bacterial translocation, increases circulatory endotoxin levels, and induces hepatic inflammation by activating the TLR4 pathway [30]. We found no significant intergroup difference in the expression levels of TLR4 or downstream proteins. However, the expression level of IL-1β was higher in Group N than in the other groups. Regarding the potential underlying mechanisms, Group N exhibited higher levels of NLRP3, p20, and NF-κB than did Group C. However, rats consuming diets containing dehulled adlay (Group NA) had an improved hepatic inflammatory status, which might have resulted from the modulation of the expression of NLRP3 and p20. One study reported that NF-κB and Nrf2 may be antagonistic to each other [31]. The activation of NF-κB may suppress the relative transcription of antioxidative genes, such as HO-1, and the expression of cAMP-response element-binding protein, affecting histone acylation associated with Nrf2 activation [32]. The activation of Nrf2 reduces the degradation of Kelch-like ECH-associated protein 1, which is associated with the activation of NF-κB [33]. Although we observed no significant intergroup differences in the TLR4 pathway, we noted that the intake of an HFHS diet reduced the diversity of the gut microbiota. Furthermore, the compositions of the gut microbiota were similar between Group N and Group NA, likely because of the adjustment of dietary fibers. Nevertheless, the relative abundances of some beneficial gut microbiota, such as *Eubacterium coprostanoligenes*, which may produce butyrate, exhibit anti-inflammatory activities, and mitigate lipid disorders [34,35], were higher in Group NA than in the other groups. The relative abundances of the potentially beneficial strains in this group correlated negatively with the hepatic levels of TG and IL-1β.

The intake of dietary fibers may prevent the development of chronic diseases; some studies have reported that whole grain fibers are more beneficial than vegetable and fruit fibers in terms of anti-inflammatory properties [7]. Although the underlying mechanisms remain unclear, bioactive components in whole grains may play important roles in mitigating inflammation. In individuals with overweight or obesity, 60 g of dehulled adlay per day was demonstrated to improve lipid profiles and inflammatory marker levels [36]. Dehulled adlay contains bioactive compounds such as dietary fibers, polyphenols, and polysaccharides, and dietary fibers may help deliver antioxidants to the gut microbiota and facilitate the production of beneficial short-chain fatty acids [37]. One study reported that dehulled adlay polysaccharides are beneficial for patients with gut metabolic disorders [38]. We previously demonstrated that a diet containing dehulled adlay can ameliorate hepatic steatosis and inflammation, but our study was limited by the different compositions of fructose in the diets of the experimental groups [39]. In the present study, the dehulled adlay sample contained 21.5% polysaccharides and 9.8 mg/gallic acid equivalents/100 g. It was incorporated into a diet containing the same amounts of total energy, macronutrients, and dietary fibers as that consumed in Group N. The dehulled adlay-supplemented diet exhibited the potential to decelerate the progression of NAFLD by ameliorating hepatic steatosis, oxidative stress, and inflammation. Furthermore, the actual daily intake amount of dehulled adlay during the experimental period was acceptable and practical in relation to the rats’ body surface area, indicating that this grain can constitute a staple food item within a diet.

## 5. Conclusions

We demonstrated that a diet supplemented with dehulled adlay may decelerate the progression of NAFLD by modulating the AMPK/Nrf2/NLRP3 inflammasome pathway and altering the gut microbiota composition. This study may serve as a reference for further clinical research and dietary modification studies.

## Figures and Tables

**Table 1:** Body composition and biochemical parameters of rats at the end of the experiment.

	C	N	NA	*p*
Weight change (g)	310.6 ± 45.7	256.9 ± 38.4	296.3 ± 45.0	0.0560
LW/BW	4.8 ± 0.8	5.0 ± 0.8	4.5 ± 0.8	0.6136
Serum				
AST (U/L)	117.0 ± 18.6	70.4 ± 23.4 *	62.1 ± 14.0 *	<0.0001
ALT (U/L)	46.5 ± 8.1	21.0 ± 8.9 *	20.5 ± 5.3 *	<0.0001
FFA (mmol/L)	0.2 ± 0.1	0.1 ± 0.1	0.1 ± 0.0	0.2724
TC (mmol/L)	1.1 ± 0.3	1.3 ± 0.3	1.1 ± 0.4	0.5755
TG (mmol/L)	0.6 ± 0.2	0.3 ± 0.1 *	0.3 ± 0.1 *	0.0007
HDL-C (mmol/L)	0.8 ± 0.2	0.9 ± 0.2	0.9 ± 0.3	0.3263
LDL-C (mmoL/L)	0.2 ± 0.0	0.3 ± 0.0	0.2 ± 0.1	0.2226
HDL-C/LDL-C	3.2 ± 0.3	3.2 ± 0.8	3.8 ± 0.7	0.0877
Hepatic cytokine				
TNF-α (pg/mg PT)	465.9 ± 192.8	640.4 ± 296.2	510.5 ± 165.2	0.2947
IL-1β (pg/mg PT)	112.9 ± 12.2	162.1 ± 52.4 *	74.78 ± 25.3 ^#^	0.0002

Data are presented in terms of the mean ± standard error of mean values; *n* = 8. One-way analysis of variance was followed by Turkey’s multiple comparison test. C, control diet; N, nonalcoholic fatty liver disease diet; NA, nonalcoholic fatty liver disease diet with dehulled adlay. Weight changes during the experimental period (final body weight minus initial body weight); LW, liver weight; BW, body weight; AST, aspartate aminotransferase; ALT, alanine aminotransferase; FFA, free fatty acid; TC, total cholesterol; TG, triglyceride; HDL, high-density lipoprotein; LDL, low-density lipoprotein; TNF-α, tumor necrosis factor-α; IL-1β, interleukin-1β; PT, protein. * *p* < 0.05 compared with Group C; ^#^ *p* < 0.05 compared with Group N.

## Data Availability

The datasets generated during the current study are available from the corresponding author upon reasonable request.

## Supplementary Materials

The following supporting information can be downloaded at: https://www.mdpi.com/article/10.3390/nu15204375/s1. Table S1: Composition of the experimental diets (g/kg diet).
