# Supplementary material for: Partial Replacement of Diet with Dehulled Adlay Ameliorates Hepatic Steatosis, Inflammation, Oxidative Stress, and Gut Dysbiosis in Rats with Nonalcoholic Fatty Liver Disease
Source: Nutrients. 2023 Oct 16;15(20):4375. doi: 10.3390/nu15204375 (PMC10610228; doi:10.3390/nu15204375)
Supplement: Supplementary file 1 [file nutrients-15-04375-s001.zip › nutrients-2618154-supplementary.pdf]

Table S1. Compositions of the experimental diets (g/kg diet).

Ingredient	N	NA
casein	258	239
L-cystine	4	4
Lodex (dextrin)	162	92
sucrose	94	94
cornstarch	-	-
lard	317	317
soybean oil	32	24
cellulose	65	60
mineral mix	65	65
vitamin mix	1	1
choline bitartrate	3	3
dehulled adlay	-	112.5

Casein high nitrogen, L-cysteine, dextrin, cellulose, mineral mixture, choline bitartrate, vitamin mixture (AIN-93VX) were acquired from MP Biomedicals, LLC (CA, USA). Dextrin-maltose was obtained from San Soon Seng Food Industries. Lard was received from President Nisshin CORP. Sucrose and soybean oil was obtained from Taiwan Sugar Corporation (Tainan, Taiwan). Dehulled adlay was obtained from Taichung district agricultural research and extension station, COA (Taichung, Taiwan). C, control diet; N, HFS diet-induced NAFLD; NA, N institute dehulled adlay diet
